# From cardiac injury to omics signatures: a narrative review on biomarkers in septic cardiomyopathy

**DOI:** 10.1007/s10238-025-01842-5

**Published:** 2025-08-21

**Authors:** Matteo Guarino, Francesco Luppi, Giacomo Maroncelli, Paolo Baldin, Anna Costanzini, Martina Maritati, Carlo Contini, Biagio Sassone, Roberto De Giorgio, Michele Domenico Spampinato

**Affiliations:** 1 Department of Translational Medicine, St. Anna University Hospital of Ferrara, Via A. Moro, 8, 44124 Cona, Ferrara, Italy; 2 Emergency Department, S. Anna University Hospital of Ferrara, Ferrara, Italy; 3 Infectious and Dermatology Diseases, St Anna University Hospital of Ferrara, Ferrara, Italy; 4 Division of Provincial Cardiology, Department of Translational Medicine, University of Ferrara, Ferrara, Italy

**Keywords:** Biomarkers, Multi-omics, Precision medicine, Sepsis, Septic cardiomyopathy

## Abstract

**Background:**

Septic cardiomyopathy (SCM) is a frequent and underdiagnosed complication of sepsis that contributes significantly to patient morbidity and mortality. Its pathophysiology involves myocardial inflammation, mitochondrial dysfunction, and microcirculatory abnormalities. Despite growing recognition, the diagnostic approach to SCM remains inconsistent, and validated biomarkers are lacking.

**Methods:**

This narrative review explores the current landscape of SCM biomarkers. PubMed, Scopus, and EMBASE were searched from inception to June 2025.

**Results:**

Traditional biomarkers are useful, but nonspecific in the septic context. Emerging biomarkers offer promising diagnostic and prognostic information, particularly in combination. Multi-omics strategies revealed transcriptomic and proteomic profiles to be potentially specific for SCM and may facilitate early detection and risk stratification. However, limitations remain in terms of standardization, assay reproducibility, and clinical translation. Composite biomarker panels and longitudinal monitoring appear to be more informative than single-point measurements.

**Conclusions:**

SCM remains a diagnostic challenge, although biomarker research is rapidly evolving. Integrating traditional and emerging biomarkers, supported by multi-omics and computational tools, may enable a shift toward precision medicine in sepsis-related cardiac dysfunction. Future efforts should focus on consensus definitions, validation in prospective cohorts, and biomarker-guided interventions to improve patient outcomes.

## Introduction

Sepsis remains one of the major concerns in global health, being responsible for over 11 million deaths annually and contributing to significant long-term morbidity among survivors [1]. Defined as a dysregulated host response to infection leading to life-threatening organ dysfunction [2, 3], sepsis represents a highly heterogeneous syndrome with complex pathophysiological mechanisms and diverse clinical phenotypes [4, 5]. Despite advances in critical care, the mortality and overall burden associated with sepsis and septic shock remain extremely high [6].

Among the various organ impairment associated with sepsis, cardiovascular dysfunction is particularly critical, being an early marker of disease severity and a driver of poor outcomes [7, 8]. Septic cardiomyopathy (SCM) is a potentially reversible myocardial dysfunction that occurs in a subset of patients with sepsis and is characterized by biventricular systolic and/or diastolic dysfunction, often in the absence of overt coronary artery disease. This dysfunction is primarily nonischemic and inflammatory in nature, driven by cytokine-induced cardiomyocyte injury, mitochondrial impairment, and microvascular disturbances, rather than traditional obstructive coronary events [9–12]. It is frequently accompanied by ventricular dilatation, reduced ejection fraction, or impaired myocardial strain, and may affect both left and right ventricles [8–15]. Despite increasing clinical and scientific interest in SCM over the past two decades, its actual epidemiology remains poorly defined. Reported prevalence rates vary widely across studies, ranging from 20% to over 60%, depending on the population studied, timing and modality of cardiac assessment, and the criteria used to define myocardial dysfunction [9, 10]. The lack of consensus on standardized diagnostic criteria, along with the dynamic nature of cardiac function in sepsis, makes it challenging to obtain reliable incidence or prevalence estimates [10]. Furthermore, SCM is often undiagnosed in daily practice, particularly in resource-limited settings or when echocardiographic evaluation is delayed or unavailable. This under-recognition contributes to unsatisfactory therapeutic opportunities and poor clinical outcomes [9, 10].

Currently, the diagnosis of SCM is largely based on echocardiographic findings and clinical evaluation, supplemented by hemodynamic measurements when available [16]. However, echocardiography is operator-dependent and may not always capture subtle or evolving myocardial dysfunction. Moreover, differentiating SCM from preexisting cardiac disease can be difficult, especially in older patients or those with underlying comorbidities such as heart failure or ischemic heart disease [12, 17]. In this context, cardiac biomarkers may provide valuable adjunctive information. Traditional biomarkers such as troponins and natriuretic peptides, i.e., brain natriuretic peptide (BNP) and N-terminal proBNP (NT-proBNP), have been widely studied in septic populations and are often elevated, but their interpretation is limited by low specificity and overlap with other causes of myocardial strain or injury [18–21]. Troponin elevation, for example, is frequently observed in sepsis and does not necessarily indicate acute coronary syndrome [18, 19], while BNP levels may reflect volume status, renal function, and systemic inflammation as much as cardiac performance [20, 21]. Moreover, there are cardiomyopathies (e.g., amyloidosis) or cardiac rhythm disorders (e.g., atrial fibrillation) that are responsible for a persistent elevation of both these biomarkers, thereby making their clinical interpretation even more challenging.

In recent years, the availability of high-throughput technologies, such as transcriptomics, proteomics, and metabolomics, provided new perspectives for biomarker discovery in sepsis-related organ dysfunction [22–25]. These approaches have led to the identification of novel candidate biomarkers that may be more closely linked to the pathophysiology of SCM, including molecules related to immune activation, endothelial injury, and mitochondrial function [26]. Additionally, integrative bioinformatics and single-cell RNA sequencing have begun to map the complex cellular and molecular landscape of sepsis-induced cardiac injury, offering a more refined view of potential diagnostic and prognostic targets [27].

This narrative review will provide a comprehensive overview of current and emerging biomarkers for septic cardiomyopathy. We will first examine the clinical utility and limitations of traditional biomarkers, then explore novel molecular markers with potential diagnostic and prognostic relevance, and finally discuss the contribution of multi-omic approaches in unveiling new biological insights. We aim at clarifying the evolving role of biomarkers in the identification and management of SCM and highlight future directions for research in this challenging field. Despite growing interest in individual biomarkers and omics-based approaches, a unified review that critically assesses and contextualizes these findings for SCM remains absent. We aim to fill this gap by integrating classical and emerging biomarker research (including multi-omic discoveries) with a focus on diagnostic challenges, translational barriers, and future clinical applications.

## Search strategy

We conducted a comprehensive literature search using PubMed, Scopus, and EMBASE from database inception to June 2025. The search strategy included the following terms: “septic cardiomyopathy” OR “sepsis-induced cardiac dysfunction” AND “biomarkers” OR “omics” OR “multi-omics” AND “diagnosis” OR “prognosis.” Additional filters were applied to include only studies involving adult human subjects. To ensure completeness, we also performed a manual search of the reference lists of relevant articles and previous reviews. Only English-language publications were considered.

## Main text

### Traditional biomarkers in septic cardiomyopathy

Cardiac biomarkers play a key role in the assessment of myocardial injury and dysfunction across a wide range of clinical settings. In the context of sepsis, however, their interpretation is particularly challenging. Sepsis induces profound changes in cardiovascular physiology, including inflammatory-driven myocardial depression, vasoplegia, and microcirculatory failure. These systemic abnormalities can lead to elevations in biomarkers commonly used to detect acute coronary syndromes or heart failure, even in the absence of primary heart disease [18–21].

In septic cardiomyopathy (SCM), traditional cardiac biomarkers, such as troponins and natriuretic peptides, are frequently elevated, but their specificity for SCM remains limited [18–21]. Moreover, sepsis itself may interfere with biomarker kinetics through mechanisms such as impaired clearance (e.g., due to renal dysfunction), altered tissue release, and systemic inflammation. Despite these limitations, these biomarkers remain widely used because of their availability, rapid half-life, and long-standing clinical familiarity [2, 3, 28].

#### Cardiac troponins

Cardiac troponins (cTnI and cTnT) are regulatory proteins of the troponin complex, which are involved in cardiomyocyte contractility. Cardiac troponin I (cTnI) and troponin T (cTnT) are both components of the troponin complex specific to cardiac muscle, but they differ in structure and assay characteristics. cTnI is exclusively expressed in cardiac tissue and is highly specific for myocardial injury, while cTnT, although also cardiac-specific, may show slight cross-reactivity in certain conditions. Both are encoded by cardiac-specific genes and are released into circulation following myocardial cell injury [29]. Troponins are released into the bloodstream following cardiomyocyte injury [29]. These markers are considered the gold standard for detecting myocardial injury [29]. Elevated troponin levels are common in patients with sepsis and have been associated with increased mortality, longer intensive care unit (ICU) stays, and greater incidence of organ dysfunction [18, 19]. However, the mechanisms underlying troponin release in sepsis are multifactorial and not necessarily linked to myocardial ischemia. Proposed mechanisms include cytokine-mediated cardiomyocyte injury, oxidative stress, mitochondrial dysfunction, and membrane leakage [18].

The diagnostic value of troponins in SCM is limited by their low specificity. Troponin elevations can occur in a variety of conditions including renal failure, pulmonary embolism, and tachyarrhythmias, all of which may coexist in septic patients [30–33]. Moreover, the degree of troponin elevation does not correlate reliably with the severity of myocardial dysfunction assessed by echocardiography [13, 16, 34]. This hampers their utility in differentiating SCM from other causes of cardiac biomarker elevation [32, 33].

Nonetheless, troponin measurements have prognostic relevance [30–35]. Several studies showed that elevated troponins in sepsis are independently associated with increased mortality, even in the absence of overt cardiac dysfunction [18, 19, 30–35]. Serial measurements may also provide dynamic information on myocardial stress or recovery, although this did not reach yet standard practice.

#### Brain natriuretic peptide and NT-proBrain natriuretic peptide

Brain natriuretic peptide (BNP) and its inactive fragment proBrain natriuretic peptide (NT-proBNP) belong to the vasoactive peptide family and are derived from the cleavage of a common precursor, proBNP, into active BNP and inactive NT-proBNP. These peptides are secreted by cardiomyocytes in response to ventricular wall stretch and volume overload. In sepsis, both markers are frequently elevated, reflecting myocardial strain and fluid shifts rather than classic heart failure [36]. Like troponins, BNP and NT-proBNP levels correlate with disease severity and prognosis, and their elevation has been linked to increased mortality and ICU length of stay [21, 37, 38].

The interpretation of natriuretic peptides in sepsis is limited by multiple confounding factors [20, 37]. Renal dysfunction, common in septic patients, impairs peptide clearance, particularly for NT-proBNP [38, 39]. Moreover, systemic inflammation and vasodilation can alter ventricular loading conditions, leading to elevated levels without overt systolic dysfunction. Studies investigating the relationship between BNP/NT-proBNP and echocardiographic findings of SCM have yielded inconsistent results [40]. Nonetheless, these biomarkers may still have a role in the early identification of septic patients at risk of cardiovascular compromise [37, 38]. When combined with echocardiographic assessment and clinical context, natriuretic peptides may contribute to risk stratification and guide fluid or vasopressor management [40].

#### Other classical markers: CK-MB, LDH, and myoglobin

Before the widespread use of cardiac troponins and natriuretic peptide assay, several other biomarkers were commonly measured to assess myocardial injury. Among these, creatine kinase-MB (CK-MB), lactate dehydrogenase (LDH), and myoglobin were historically considered part of the standard cardiac panel. However, their utility in SCM is now limited due to low specificity, poor sensitivity, and significant overlap with other causes of systemic illness [41].

CK-MB is an isoenzyme predominantly found in cardiac muscle, but it is also present in skeletal muscle. It plays a role in cellular energy metabolism and is released into the bloodstream following muscle cell damage. In sepsis, CK-MB levels may rise due to global muscle injury, rhabdomyolysis, or increased cellular turnover, making it an unreliable indicator of myocardial-specific damage. Its shorter half-life and susceptibility to noncardiac influences further diminish its diagnostic value in the septic context [41, 42].

LDH is a ubiquitous intracellular enzyme released during cell lysis and tissue injury. While elevated LDH may reflect ongoing cellular damage in sepsis, it lacks organ specificity and does not provide actionable information about cardiac involvement [42, 43]. Its levels are influenced by hemolysis, hepatic dysfunction, and malignancies, all of which may coexist with sepsis.

Myoglobin, a heme protein released from both cardiac and skeletal muscle, rises rapidly after injury, but is cleared quickly through the kidneys. In septic patients, myoglobin levels can be elevated due to noncardiac muscle damage or renal impairment, limiting its interpretability. Its lack of specificity and rapid kinetics make it largely obsolete in modern critical care settings [43].

Although these markers may still be listed in some laboratory panels, especially in resource-limited settings, their clinical relevance in the diagnosis or prognostication of SCM is minimal. They are not recommended as stand-alone indicators of myocardial dysfunction and should not guide clinical decision-making in isolation.

### Emerging biomarkers in septic cardiomyopathy

Recent advances in molecular biology and high-throughput technologies have led to the discovery of a growing number of novel markers with potential applications in the diagnosis, monitoring, and prognostication of SCM [44, 45]. These emerging indicators reflect various pathophysiological pathways, including inflammation, oxidative stress, myocardial stretch, endothelial activation, and immune dysregulation. Unlike traditional biomarkers, which largely indicate cardiac injury or wall stress, many of these new markers aim to capture the early and dynamic processes of septic myocardial dysfunction [8, 46]. The main promise of these novel markers lies in their potential for earlier detection of SCM, improved prognostic accuracy, and, in some cases, mechanistic insights that could support therapeutic targeting [46]. However, most of them remain confined to the research setting, with limited validation in large-scale, multicenter cohorts. Additionally, significant heterogeneity in study design, timing of sampling, assay platforms, and echocardiographic definitions of SCM hampered their clinical translation.

In the following sections, we review the most relevant and promising emerging biomarkers, with a focus on their biological rationale, current evidence, and limitations in the context of SCM (further summarized in Table 1).

**Table 1:** Overview of emerging biomarkers in septic cardiomyopathy

Name	Acronym	Function/pathway	Pros	Cons	Clinical evidence level
Secretoneurin [47–49]	–	Calcium regulation, endothelial activation	Novel pathway; early rise	Limited availability; minimal validation	Experimental
Soluble ST2 [50–56]	sST2	IL-33 receptor decoy, inflammation	Strong prognostic signal; less confounded	Nonspecific; expensive	Moderate
Presepsin [56–62]	PSP sCD14-ST	Monocyte activation, innate immunity	Early sepsis detection; good NPV	Noncardiac; affected by renal function	Moderate
Procalcitonin [63, 64]	PCT	Systemic inflammation, bacterial burden	Available; widely used	No direct link to myocardial injury	Routine, nonspecific
Mid-regional pro-adrenomedullin [65–68]	MR-proADM	Endothelial stress, vascular dysfunction	Prognostic value; reflects cardiovascular strain	Not cardiac-specific; limited SCM studies	Moderate to low
Heart-type fatty acid-binding protein [69–72]	H-FABP	Early myocardial injury marker	Early detection; fast kinetics	Renal dependence; limited validation	Low to moderate
Calprotectin [73, 74]	S100A8/A9	Neutrophil-derived alarmin; systemic inflammation via TLR4/RAGE	Early release; correlates with sepsis severity; promising experimental cardioprotective effects	Low cardiac specificity; not SCM-specific; limited validation	Low
Extracellular vesicles [91]	EVs	Intercellular signaling, immune modulation, myocardial stress response	Reflect cellular status; detectable via liquid biopsy; multi-analyte potential	No standardized isolation; heterogeneous cargo	Low to moderate
Cell-free mitochondrial DNA [92, 93]	cf-mtDNA	Innate immune activation via TLR9; stress-linked DAMP	Early release; correlates with severity; measurable with simple assays	Low specificity; unclear tissue origin	Low

cf-mtDNA, Cell-free mitochondrial DNA; DAMP, damage-associated molecular pattern; EV, extracellular vesicle; H-FABP, heart-type fatty acid-binding protein; IL-33, interleukin 33; MR-proADM, mid-regional pro-adrenomedullin; NPV, negative predictive value; PCT, procalcitonin; PSP/SCD14-ST, presepsin; RAGE, receptor for advanced glycation end-products; SCM, septic cardiomyopathy; sST2, soluble ST2; TLR, Toll-like receptors

#### Secretoneurin

Secretoneurin is a neuropeptide derived from chromogranin/secretogranin proteins, produced by neuroendocrine and cardiac cells, and involved in calcium signaling, inflammation, and endothelial function. It has emerged as a potential cardiovascular biomarker, particularly in critically ill patients, where elevated levels have been associated with increased mortality, arrhythmia risk, and myocardial dysfunction [47–49].

In sepsis, secretoneurin levels appear to correlate with disease severity and may reflect early cardiac involvement. A few studies have shown that it rises in parallel with troponins and BNP, but may offer additional prognostic value due to its distinct pathophysiological signature. Importantly, it may act as both a marker and a mediator of myocardial stress via calcium dysregulation and endothelial activation [47, 48]. However, clinical data on secretoneurin in SCM remain limited. The marker has not yet been validated for routine use, and compared to established biomarkers its performance is still under investigation.

#### Soluble suppression of tumorigenicity-2 (sST2)

sST2 is a member of the interleukin-1 receptor family, released by cardiomyocytes and immune cells in response to mechanical strain and inflammation [50]. It acts as a decoy receptor for IL-33, disrupting cardioprotective signaling and promoting myocardial remodeling and fibrosis. In heart failure, sST2 is a well-established prognostic biomarker, included in some risk stratification algorithms [51].

In sepsis, sST2 levels are frequently elevated and correlate with both disease severity and mortality. Several studies have suggested that sST2 may help identify patients at risk for septic cardiomyopathy, particularly when used in combination with echocardiographic assessment. Unlike natriuretic peptides, sST2 is less affected by age, body mass, and renal function, which may confer an advantage in critically ill populations [52–56].

Nevertheless, its role as a specific marker of SCM remains to be validated. The lack of cardiac specificity and its modulation by systemic inflammation may limit its stand-alone diagnostic value.

#### Presepsin (PSP)

PSP, also known as soluble CD14 subtype (sCD14-ST), is a fragment released by monocytes and macrophages during bacterial phagocytosis. It is part of the innate immune response and belongs to the pattern recognition receptor family [56]. It has been widely studied as an early diagnostic marker for sepsis and as a potential prognostic tool [56–59].

While not specific to cardiac dysfunction, some studies have found associations between PSP levels and myocardial depression in septic patients [60]. These findings suggest that it may reflect the interplay between innate immune activation and organ dysfunction, including the heart [60–62].

However, its utility in identifying SCM is likely indirect. The elevation of PSP reflects the septic state itself rather than myocardial-specific pathophysiology. It may play a complementary role when interpreted in conjunction with cardiac biomarkers and imaging.

#### Procalcitonin (PCT)

Procalcitonin is a prohormone of calcitonin, normally produced by thyroid C cells but also expressed in various tissues during systemic bacterial infection [63]. It is a widely used marker of bacterial infection and systemic inflammation, especially in guiding antibiotic therapy [63]. It is not a cardiac biomarker per se but is frequently included in sepsis panels. Some studies have examined the relationship between PCT levels and cardiac dysfunction in sepsis, with conflicting results [64]. While elevated PCT may reflect disease severity and correlate with worse outcomes, it does not provide direct information about myocardial involvement [56, 63, 64]. Moreover, it is heavily influenced by infection burden, renal function, and timing of measurement [63].

In the context of SCM, PCT may serve as a general marker of systemic burden but lacks the specificity or mechanistic relevance needed for targeted cardiac assessment.

#### Mid-regional pro-adrenomedullin (MR-proADM)

Adrenomedullin is a vasodilatory peptide with roles in endothelial barrier function, vascular tone, and microcirculatory integrity. MR-proADM is a stable surrogate for biologically active adrenomedullin and is gaining attention as a prognostic marker in sepsis [59, 65, 66].

In patients with septic shock, elevated MR-proADM levels correlate with endothelial dysfunction, organ failure, and mortality. Recent data suggest a potential link with myocardial dysfunction. This may be mediated through microvascular hypoperfusion and capillary leak. [67, 68].

Though not cardiac-specific, MR-proADM may reflect global cardiovascular stress and hemodynamic instability. Its role in SCM is still exploratory but potentially valuable, particularly when integrated into multimodal risk scores.

#### Heart-type fatty acid-binding protein (H-FABP)

H-FABP is a small cytoplasmic protein abundant in cardiomyocytes, part of the fatty acid-binding protein family, released rapidly after myocardial injury. Its kinetics are faster than troponin, making it attractive for early detection of cardiac damage [69].

In sepsis, elevated H-FABP levels have been associated with myocardial dysfunction and increased mortality [70, 71]. It may outperform troponins in identifying early myocardial involvement, especially when troponin levels remain within the normal range [67, 72]. However, its interpretation can be challenging due to renal clearance and possible elevation from noncardiac muscle injury.

Although promising, H-FABP is not yet widely available and lacks standardized thresholds for SCM.

#### Calprotectin (S100A8/A9)

Calprotectin is a neutrophil-derived alarmin released early in the inflammatory cascade, with plasma levels > 2–3 mg/L shown to differentiate sepsis from sterile inflammation and correlate with illness severity and multi-organ dysfunction [73]. While its role as a general sepsis biomarker is well supported by recent studies, evidence specific to cardiac involvement remains limited. A single-center cohort reported an association between high calprotectin levels and left ventricular systolic dysfunction [74], and in experimental models, pharmacologic inhibition or gene knockout of S100A8/A9 reduced myocardial depression and inflammation [73]. The proposed mechanism involves TLR4/RAGE signaling and mitochondrial dysfunction, but its direct cardiotropic role in humans remains unproven. Although mechanistically intriguing, calprotectin lacks cardiac specificity and validated thresholds for SCM.

### Multi-omics and bioinformatics approaches

In recent years, the advancement of high-throughput molecular profiling technologies has revolutionized our understanding of complex syndromes such as sepsis and its cardiovascular complications [75–80]. SCM may benefit significantly from multi-omics investigations, which enable comprehensive characterization at genomic, transcriptomic, proteomic, and metabolomic levels [77–80]. Multi-omics approaches can unravel molecular patterns that are undetectable by conventional methods, revealing novel biomarkers and therapeutic targets [77]. By integrating large-scale datasets from bulk and single-cell RNA sequencing, proteomics, and metabolomics with clinical phenotypes, researchers have begun to identify candidate genes and proteins associated with myocardial dysfunction during sepsis [76]. Recent metabolomic studies have identified several promising biomarker candidates in SCM, particularly those involved in mitochondrial energy metabolism and oxidative stress. For example, abnormal acylcarnitine succinate and lactate levels have been reported in patients with SCM, suggesting impaired fatty acid oxidation and mitochondrial overload [26, 77]. Similarly, glutamine depletion and branched-chain amino acid dysregulation have emerged as potential indicators of metabolic stress and immune dysfunction in SCM [25, 26, 77]. These findings highlight the value of metabolomics in identifying subtle biochemical shifts associated with early cardiac involvement, potentially enabling more timely and targeted interventions. When integrated with transcriptomic and proteomic data, metabolomics may help delineate molecular endotypes of SCM, offering a path toward patient-specific diagnostic and therapeutic strategies. Moreover, bioinformatic tools, such as network analysis, pathway enrichment, and machine learning-based clustering, are increasingly employed to distinguish SCM-specific molecular signatures [76]. These integrative frameworks may pave the way for future diagnostic algorithms, risk stratification tools, and personalized interventions in septic patients with cardiac involvement. Recent advances in artificial intelligence (AI) and machine learning (ML) have further enhanced the power of multi-omics integration in SCM research. Supervised models, such as random forests, support vector machines, and least absolute shrinkage and selection operator (LASSO) regression, have been employed to identify key features among thousands of genes, transcripts, and proteins, improving diagnostic and prognostic prediction [79, 80]. Unsupervised approaches, such as integrative clustering and consensus k-means, have been used to delineate molecular subtypes of SCM with distinct inflammatory and metabolic signatures [79]. In addition, deep learning-based frameworks are beginning to emerge, being capable of handling large-scale multi-omics data to uncover nonlinear relationships and hidden patterns [79]. These tools support the development of precision medicine strategies and may ultimately guide personalized interventions in septic patients with myocardial dysfunction. A notable example of omics-driven translational insight in SCM is the identification of STAT3 as a key transcriptional regulator linking inflammatory signaling and mitochondrial dysfunction. Integrated transcriptomic analyses have consistently shown STAT3 dysregulation in septic myocardial tissue [80, 81]. Preclinical studies using murine models demonstrated that cardiomyocyte-specific modulation of STAT3 activity can attenuate myocardial inflammation and improve cardiac output, with ginsenoside-Rc and other compounds showing promising effects via the STAT3/FoxO3a/SIRT1 axis [82, 83]. These findings suggest that omics-guided target discovery may lay the ground for future therapeutic strategies in SCM.

Below, we discuss key molecular candidates recently proposed as potential biomarkers or mechanistic drivers of SCM, as identified by multi-omics studies (summarized in Table 2).

**Table 2:** Multi-Omics-Derived Biomarkers in Septic Cardiomyopathy

Gene/protein	Pathway/function	Evidence type	Pros	Cons	Clinical readiness
STAT3 [81–83]	Mitochondrial function, inflammation regulation	Transcriptomics (murine + human), functional studies	Mechanistic link to cardiomyocyte protection; consistent upregulation	Not directly measurable in serum; intracellular	Experimental
CCL2 (MCP-1) [83–85]	Monocyte recruitment, endothelial activation	Transcriptomics, plasma proteomics, clinical correlation	Measurable in plasma; strong inflammatory signal	Poor cardiac specificity; confounded by systemic inflammation	Moderate
MYC [83–85]	Metabolic reprogramming, mitochondrial dysfunction	Transcriptomics (bulk RNA-seq), pathway enrichment	Central metabolic hub; conserved across species	Pleiotropic; oncogenic concerns; non-circulating	Experimental
SERPINE1 (PAI-1) [83, 85, 86]	Fibrinolysis inhibition, endothelial dysfunction	Proteomics, clinical plasma data	Links coagulation, endothelium, and perfusion; measurable	Nonspecific; elevated in many critical illnesses	Moderate
LGALS3 (Galectin-3) [53, 56]	Inflammation, fibrosis, remodeling	Single-cell RNA-seq, integrative clustering	Involved in cardiac fibrosis; measurable; known in HF	Not SCM-specific; confounded by CKD, aging	Moderate
TIMP1 [88]	Matrix remodeling, anti-MMP activity	scRNA-seq, proteomics	Reflects myocardial remodeling; linked to severity	Variable expression; influenced by other factors	Experimental
FN1 (Fibronectin-1) [89]	ECM structure, integrin signaling	Single-cell RNA-seq, network analysis	Key player in myocardial injury response	Very poor specificity; ubiquitous expression	Experimental

CKD, chronic kidney disease; ECM, extracellular matrix; FN1, fibronectin-1; PAI-1, plasminogen activator inhibitor-1; C–C motif chemokine ligand 2; MYC, myelocytomatosis oncogene; MMP, matrix metalloproteinases; LGAL3, galectin-3; STAT3, signal transducer and activator of transcription 3; RNA, ribonucleic acid; scRNA, small conditional ribonucleic acid; SCM, septic cardiomyopathy; TIMP1, tissue inhibitor of metalloproteinases 1

#### STAT3 (signal transducer and activator of transcription 3)

STAT3 is a transcription factor activated by a wide range of cytokines and growth factors, playing a crucial role in cell survival, mitochondrial function, and immune regulation [80]. Transcriptomic studies in septic myocardial tissue and cardiomyocytes exposed to endotoxins have consistently shown STAT3 dysregulation [43, 44].

In murine models, cardiomyocyte-specific STAT3 activation appears to confer protection against inflammatory injury, while its inhibition worsens cardiac dysfunction. In humans, increased STAT3 expression has been linked to septic shock and worse cardiac performance, suggesting both diagnostic and mechanistic relevance [81–83].

#### CCL2 (C–C motif chemokine ligand 2/MCP-1)

CCL2 is a chemokine involved in monocyte recruitment and endothelial activation. Upregulated in both transcriptomic and proteomic datasets from septic hearts, CCL2 is associated with inflammation-induced cardiac remodeling [44].

Elevated circulating levels of CCL2 have been observed in patients with septic shock and correlate with disease severity [83–85]. Some evidence links CCL2 overexpression to microvascular dysfunction and myocardial depression, although cardiac specificity is limited.

#### MYC (MYC proto-oncogene, bHLH transcription factor)

The MYC gene is a central regulator of cell proliferation and metabolic reprogramming [80]. RNA-seq analyses of septic myocardium and bioinformatic clustering have highlighted MYC as a potential hub gene linked to cardiac metabolic derangement [83, 85].

MYC upregulation in sepsis may contribute to impaired mitochondrial bioenergetics and maladaptive cardiac remodeling [85]. However, the interpretation of MYC expression is complex due to its pleiotropic effects and role in various stress responses.

#### SERPINE1 (plasminogen activator inhibitor-1, PAI-1)

SERPINE1 is a key regulator of fibrinolysis and endothelial homeostasis [78]. Proteomic studies have identified increased PAI-1 levels in patients with severe sepsis and cardiovascular failure [78, 83, 86, 87]. Its expression correlates with both coagulopathy and tissue hypoperfusion, which are common features in SCM.

In cardiac tissue, SERPINE1 may contribute to micro-thrombosis, capillary leakage, and impaired oxygen delivery. High levels have also been associated with worse outcomes in heart failure and post-cardiac surgery patients, reinforcing its relevance in cardiovascular stress [88].

#### Other candidates from single-cell and integrated analyses

Single-cell RNA-seq studies have recently provided new insights into cell-type–specific responses in the septic heart [76, 88]. Endothelial cells, fibroblasts, and immune infiltrates show distinct transcriptional alterations during septic shock. Candidate genes, such as LGALS3 (galectin-3), TIMP1, and FN1, have emerged from integrated analyses as potential contributors to myocardial inflammation and remodeling [53, 56, 89, 90].

These approaches also allow for the discovery of gene modules that correlate with echocardiographic parameters or hemodynamic data, thus enabling functional annotation of molecular signatures. Machine learning applied to multi-omic datasets has shown promise in predicting left ventricular dysfunction in sepsis, although external validation is still lacking [79].

#### Emerging liquid biopsy signals: extracellular vesicles and cell-free mitochondrial DNA

In parallel with traditional multi-omics pipelines, liquid biopsy strategies, such as the analysis of circulating extracellular vesicles (EVs) and cell-free mitochondrial DNA (cf-mtDNA), are gaining interest in cardiovascular and critical care research. EVs are nanometer-sized vesicles secreted by cells under stress or injury, and their molecular cargo (comprising proteins, lipids, and nucleic acids) can be profiled through proteomics and miRNA-sequencing, aligning them with omics-based frameworks. Studies have shown that EVs originating from inflammatory or endothelial sources may mirror myocardial strain and immune dysregulation in septic patients, potentially serving as dynamic indicators of disease progression [91].

Furthermore, cf-mtDNA is released following cellular stress or damage and functions as a damage-associated molecular pattern (DAMP), triggering immune responses via Toll-like receptor 9 (TLR9). Elevated plasma cf-mtDNA levels have been correlated with poor outcomes in sepsis and myocardial injury [92, 93]. While its detection is not strictly omics-driven, cf-mtDNA represents a promising early biomarker of systemic and cardiac stress, especially when integrated within computational models or composite biomarker panels. Together, EVs and cf-mtDNA expand the molecular armamentarium available for risk stratification and early recognition of SCM. Since EVs and cf-mtDNA have been added as emerging noninvasive biomarkers, reflecting myocardial stress and systemic inflammation in sepsis, they have been added to Table 1.

### Integration and clinical application of biomarkers in septic cardiomyopathy

The identification of reliable biomarkers for septic cardiomyopathy (SCM) has opened promising avenues for improving the clinical management of this often-underdiagnosed complication of sepsis [83]. However, translating these molecular signals into actionable tools at the bedside remains challenging. A nuanced integration of biomarker data with clinical, imaging, and hemodynamic parameters could potentially enhance diagnostic accuracy, prognostic stratification, and therapeutic decision-making in patients with SCM. Yet, to fully realize the potential of precision medicine, it is critical to account for patient-specific variables (such as age, sex/gender, and pharmacologic profiles) that modulate biomarker levels and affect interpretation in SCM. Age-related changes (including reduced glomerular filtration, altered vascular compliance, and myocardial remodeling) can affect clearance and baseline levels of biomarkers, e.g., NT-proBNP and troponins, often necessitating age-adjusted thresholds [17–21]. Similarly, sex-based biological differences play a role: female patients may exhibit cardioprotective responses mediated by estrogens, while men and women show distinct expression patterns of stress-associated microRNAs (e.g., miR-1, miR-21, miR-146a) [27, 76, 91]. Moreover, medications commonly used in septic or cardiac patients (e.g., beta-blockers, renin angiotensin aldosterone system inhibitors, steroids) can modulate biomarker release or interfere with inflammatory pathways, complicating diagnostic interpretation. Addressing these variables is essential to ensure the clinical utility of biomarkers within a precision medicine framework, and future studies should systematically account for these modifiers to refine diagnostic and prognostic algorithms.

#### Early identification and diagnostic utility

Diagnosing SCM in its early stages is notoriously difficult, as the syndrome lacks pathognomonic signs and often overlaps with other cardiac and extracardiac causes of hemodynamic instability [16]. Conventional tools such as transthoracic echocardiography, while useful, may not capture subtle myocardial dysfunction or distinguish between sepsis-induced changes and preexisting abnormalities [16, 17, 34]. In this context, biomarkers can provide timely biochemical insights into myocardial stress, injury, or inflammation, often before overt clinical deterioration occurs [43, 44].

Cardiac troponins and natriuretic peptides are widely available and frequently used in the critical care setting. Their elevation may raise suspicion for myocardial involvement in septic patients, particularly when supported by suggestive echocardiographic findings [18–21, 34]. However, their diagnostic specificity remains suboptimal, as both can be influenced by renal dysfunction, preexisting cardiac disease, and systemic inflammation. Emerging biomarkers, such as secretoneurin, sST2, or heart-type fatty acid-binding protein (H-FABP), appear more tightly linked to mechanisms specific to SCM, including myocardial strain, endothelial dysfunction, and innate immune activation [47, 48, 50–56, 69, 70]. When interpreted in combination, these biomarkers may increase diagnostic confidence and help delineate SCM from other causes of circulatory failure.

The integration of biomarker profiles into clinical algorithms or decision-support tools (particularly when combined with echocardiographic markers such as global longitudinal strain) may allow for earlier recognition of SCM, potentially enabling earlier hemodynamic optimization or cardioprotective interventions.

#### Prognostic stratification and risk assessment

Beyond diagnosis, biomarkers play a pivotal role in prognostic stratification. Numerous studies have demonstrated associations between elevated levels of troponin, BNP, NT-proBNP, and adverse outcomes in septic patients, including higher mortality, longer durations of mechanical ventilation, increased vasopressor requirements, and prolonged ICU stays [18–21]. While these associations are not specific to SCM, they suggest that biomarker elevations reflect the severity of organ dysfunction, including myocardial compromise.

More novel biomarkers, such as sST2, galectin-3, TIMP1, and secretoneurin, have shown promise in predicting adverse outcomes more specifically related to cardiac dysfunction in sepsis [47–56, 89]. Likewise, omics-derived biomarkers, such as MYC, STAT3, and CCL2, have been associated with transcriptional and inflammatory profiles consistent with myocardial injury and systemic immune dysregulation [42, 81–84]. These findings suggest that a multimodal biomarker approach may allow clinicians to identify patients at highest risk for sustained or irreversible myocardial dysfunction, potentially guiding the intensity of monitoring and therapeutic escalation.

Beyond predictive biomarkers, emerging omics data suggest that endothelial-derived molecules, such as SERPINE1, angiotensin II (Ang-II), and von Willebrand factor (vWF), may constitute mechanistic drivers of myocardial dysfunction in SCM. Sepsis-induced endotheliopathy has been increasingly recognized as a central contributor to inflammation-driven coagulopathy and cardiac damage. Elevated SERPINE1 levels are associated with impaired fibrinolysis and correlate with systemic inflammation and cardiovascular failure [78, 83, 86, 87]. Notably, SERPINE1 and endothelial-derived vWF often show coordinated upregulation in septic patients, indicating a convergent molecular axis between endothelial stress and thrombo-inflammatory signaling. Angiotensin II, a vasoconstrictive and pro-inflammatory peptide, further amplifies myocardial damage by inducing oxidative stress via NADPH oxidase (NOX)-mediated reactive oxygen species generation, and has been shown to exacerbate cardiac injury in experimental sepsis [94]. Recent in vitro findings suggest that downregulation of vWF may attenuate Ang-II-induced endothelin-1 (ET-1) upregulation by mitigating NOX-mediated superoxide production [95]. In parallel, circulating biomarkers, such as mid-regional pro-adrenomedullin (MR-proADM) and pro-endothelin-1 (proET-1), have been linked to myocardial injury in septic shock, highlighting the endothelial–cardiac interface as a prognostically relevant domain [96]. Taken together, these interactions underscore a complex network of endothelial dysfunction, coagulation imbalance, and oxidative stress, with SERPINE1/Ang-II/vWF emerging as promising therapeutic and prognostic targets in SCM.

In clinical practice, the availability of reliable prognostic biomarkers could inform early decisions regarding the need for advanced monitoring, the suitability for aggressive hemodynamic support, or even the candidacy for extracorporeal life support in selected patients.

#### Therapeutic guidance and monitoring

Although the use of biomarkers to guide therapy in SCM remains largely investigational, emerging evidence suggests that certain markers may assist in tailoring therapeutic strategies. For instance, trends in NT-proBNP or MR-proADM may help guide fluid resuscitation and inotropic support, providing feedback on myocardial filling pressures and vascular tone, respectively [20, 59, 65–68]. Similarly, markers such as sST2 or galectin-3, which reflect myocardial stress and fibrosis, could identify patients who might benefit from anti-inflammatory or anti-fibrotic approaches, though such interventions remain experimental in this setting [50–56]. Serial biomarker measurements could also offer a dynamic view of disease trajectory, potentially distinguishing transient dysfunction from progressive myocardial injury [67]. This approach could support the timing of therapeutic interventions, assess response to treatment, and contribute to decisions regarding de-escalation of support.

To move toward biomarker-guided therapy, further studies are required to validate specific cutoff values, evaluate treatment response curves, and determine the additive value of biomarker monitoring in existing clinical algorithms.

#### Challenges in clinical translation

Despite these potential benefits, several barriers currently hinder the routine clinical application of SCM biomarkers. One major limitation is the lack of standardized diagnostic criteria for SCM, which complicates the interpretation and comparison of biomarker studies [25, 44]. Moreover, many biomarkers lack well-defined thresholds specific to sepsis-related myocardial dysfunction, and their levels may be confounded by factors such as age, renal impairment, preexisting cardiovascular disease, and the systemic inflammatory milieu.

In addition, the biological variability of several markers and the absence of longitudinal data in most studies make it difficult to determine their true predictive value over time. While some assays (like troponins and BNP) are readily available and affordable, others remain expensive, technically demanding, or inaccessible in routine critical care settings. Even for emerging biomarkers with promising data, their lack of validation in large, multicenter cohorts limits their applicability [45].

Ultimately, for biomarkers to be integrated into the clinical workflow of septic patients, they will need to be embedded within robust, evidence-based algorithms that consider not only their individual characteristics but also their performance in combination with other clinical, imaging, and physiological data.

### Limitations of current biomarker research

Despite the growing body of literature exploring the role of biomarkers in SCM, the current state of research remains fragmented and burdened by significant limitations. These challenges hinder not only the clinical applicability of available data but also the generalizability and reproducibility of study findings. Understanding the pitfalls of current research is essential to designing more rigorous investigations and building a framework for the future clinical integration of biomarkers [16].

One of the most fundamental limitations lies in the absence of a universally accepted definition of SCM. The lack of clear diagnostic criteria, standardized echocardiographic parameters, and consistent clinical endpoints results in considerable heterogeneity among studies [97]. Some investigations define SCM based on left ventricular systolic dysfunction, while others include diastolic impairment or right ventricular failure; still others rely solely on elevated cardiac biomarkers without confirmatory imaging [46]. This variability severely undermines the comparability of results across studies and complicates the identification of biomarker patterns specific to SCM as opposed to general cardiovascular or septic phenomena.

Study populations are also often heterogeneous and underpowered. Many investigations include small sample sizes, lack control groups, or include patients with a wide range of preexisting cardiac conditions, making it difficult to isolate the effects of sepsis on myocardial function. Furthermore, key confounding variables, such as chronic kidney disease, diabetes, atrial fibrillation, and age-related cardiac remodeling, are frequently underreported or insufficiently adjusted for. This contributes to signal dilution and limits the strength of any proposed associations [98, 99].

Another major limitation is the predominance of single time-point biomarker measurements. Most studies collect biomarker data at admission or within the first 24 h of ICU stay, without tracking dynamic changes over time. This static approach fails to capture the temporal evolution of SCM, which is often characterized by a biphasic or fluctuating course. As a result, the prognostic and therapeutic value of serial measurements remains largely unexplored, despite its potential clinical relevance [72, 100].

In addition, most of the available biomarker studies are observational in design and lack interventional validation. While associations between biomarker levels and outcomes have been reported repeatedly, few studies have tested whether biomarker-guided interventions can improve clinical endpoints such as mortality, organ dysfunction, or ICU length of stay. Without such interventional evidence, biomarkers remain diagnostic adjuncts rather than active tools in clinical decision-making [47–56, 69–90].

The translation of omics-derived biomarkers poses further challenges. Although high-throughput technologies have identified numerous candidate molecules (ranging from transcription factors to signaling peptides) most have yet to be validated in independent cohorts or implemented in clinically feasible assays. The complexity, cost, and turnaround time of omics platforms also limit their integration into real-time bedside decision-making, especially in resource-constrained environments.

Finally, publication bias and the selective reporting of positive results further distort the landscape. Studies demonstrating significant associations are more likely to be published, while negative or inconclusive findings remain underrepresented. This skews meta-analytic estimates and overstates the apparent utility of certain biomarkers, reinforcing a cycle of overinterpretation and premature clinical enthusiasm.

**Fig. 1:**
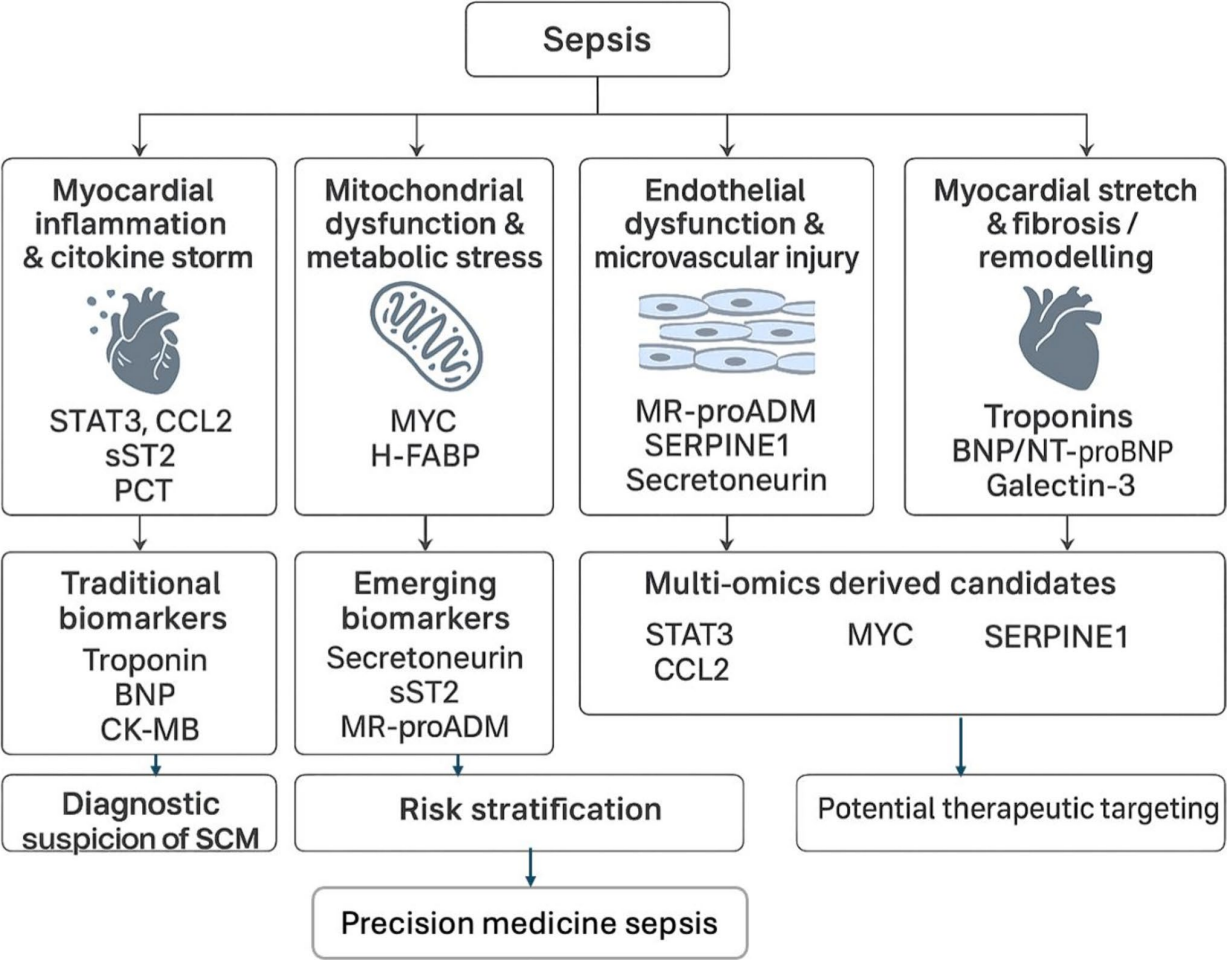
Mechanistic pathways contributing to septic cardiomyopathy and associated biomarkers. Traditional, emerging, and multi-omics-derived biomarkers are linked to distinct pathophysiological processes, supporting a precision medicine approach

To address these limitations, future research will need to adopt more standardized definitions of SCM, enroll larger and more homogeneous cohorts, incorporate serial biomarker measurements, and focus on prospective, interventional designs. Only through such methodological rigor will the true potential of biomarkers in SCM be realized (Fig. 1).

## Future perspectives and research directions

The field of SCM biomarker research stands at a critical juncture. While significant progresses have been made in identifying both traditional and emerging biomarkers, the road toward clinical translation remains long and complex [97, 98]. The integration of multidisciplinary approaches, technological innovations, and rigorous study designs will be essential to transform biomarker discovery into clinical impact [101].

A crucial first step will be to establish a shared, operational definition of SCM. Consensus criteria (ideally based on a combination of echocardiographic parameters, biomarker thresholds, and clinical context) would allow for harmonization across studies and improve both comparability and reproducibility [34, 98]. International collaborations, akin to those seen in the Sepsis-3 initiative, could play a central role in this effort by fostering standardization and data sharing.

From a biomarker development standpoint, the future lies in integrative, multi-marker strategies rather than reliance on single molecules [83]. The complexity of SCM pathophysiology (encompassing myocardial inflammation, metabolic dysregulation, mitochondrial dysfunction, and microvascular alterations) calls for composite panels that reflect multiple biological axes [99]. Coupling classical markers such as troponins or natriuretic peptides with novel candidates like sST2, PSP, or transcriptomic signatures may enhance both sensitivity and specificity [83]. Machine learning algorithms could be instrumental in identifying the most predictive biomarker combinations and in developing risk stratification tools tailored to individual patients [79].

Longitudinal biomarker profiling is another promising frontier. By capturing dynamic changes in biomarker levels over the course of illness, future studies may be able to delineate temporal patterns associated with SCM onset, progression, or resolution [98]. This would allow not only earlier diagnosis but also the identification of therapeutic windows for targeted interventions. The development of point-of-care technologies capable of rapid serial biomarker assessment will be pivotal in this context.

Another direction involves bridging the gap between discovery and clinical implementation. Translational pipelines must prioritize biomarker candidates that are not only biologically relevant but also feasible for bedside use (robust, cost-effective, reproducible, and compatible with existing laboratory infrastructure). Moreover, regulatory pathways for biomarker approval and clinical adoption need to be clarified and streamlined to facilitate uptake [100].

Importantly, future research must move beyond associative findings toward interventional validation. Randomized controlled trials that stratify patients based on biomarker profiles and test biomarker-guided therapeutic strategies will be critical to demonstrating clinical utility. For example, titrating inotropic support, immunomodulatory therapy, or fluid management based on biomarker trends may offer a precision medicine approach to SCM care. These trials should be designed with adequate power, stratification by sepsis severity and comorbidities, and clearly defined endpoints.

The incorporation of multi-omics platforms (including transcriptomics, proteomics, metabolomics, and even microbiomics) will undoubtedly enrich the biomarker landscape [26, 77, 79, 80]. These approaches offer unparalleled resolution of the molecular underpinnings of SCM, revealing novel pathways and therapeutic targets. However, their real-world implementation will require advances in computational biology, data integration, and model interpretability to ensure that findings translate into actionable clinical insights.

Finally, large-scale, multicenter biomarker registries, shared repositories of omics data, and open-access machine learning models could serve as accelerators for discovery and validation. Multidisciplinary collaboration between intensivists, cardiologists, molecular biologists, and data scientists will be key to sustaining innovation.

## Conclusions

SCM remains a clinically elusive yet pathophysiologically distinct manifestation of sepsis-associated organ dysfunction. Despite its high prevalence and strong association with adverse outcomes, SCM is still underrecognized, partly due to the lack of standardized diagnostic criteria and validated biomarkers. In recent years, considerable progress has been made in understanding its underlying mechanisms, and biomarker research has emerged as a promising avenue to bridge the gap between pathophysiology and clinical management.

Traditional markers, such as troponins and natriuretic peptides, have laid the foundation for early recognition of myocardial involvement in sepsis, but their limited specificity and diagnostic granularity have prompted the search for more nuanced indicators. Emerging biomarkers (including sST2, secretoneurin, PSP, and MR-proADM) offer insight into specific pathogenic axes such as inflammation, myocardial stress, endothelial dysfunction, and immune activation. The recent application of multi-omics and systems biology approaches has further enriched this landscape, highlighting novel molecular signatures and pointing toward more integrated, patient-specific diagnostic strategies.

Nevertheless, substantial challenges remain before biomarkers can be fully integrated into routine care. Analytical variability, the need for contextual interpretation, and the lack of prospective validation across diverse populations hinder widespread adoption. Future directions must prioritize harmonization of definitions, longitudinal assessment of biomarker dynamics, and the development of composite panels that reflect the multifactorial nature of SCM. Clinical trials stratified by biomarker profiles will be essential to determine whether precision-guided interventions can meaningfully impact outcomes.

Finally, the integration of biomarkers into the diagnostic and therapeutic paradigm of SCM holds the potential to refine risk stratification, personalize treatment, and improve prognostication in critically ill patients. This transition from descriptive recognition to mechanistic and biomarker-informed management may represent a paradigm shift in how we approach myocardial dysfunction in sepsis, an evolution long overdue and urgently needed. The time has come to validate and implement a multi-marker strategy for SCM diagnosis and management, moving from retrospective association to prospective clinical action.

## Data Availability

No datasets were generated or analysed during the current study.

## Abbreviations

AI: Artificial intelligence
Ang-II: Angiotensin II
bHLH: Basic helix–loop–helix
BNP: Brain natriuretic peptide
CCL2: C–C motif chemokine ligand 2
CD14: Cluster of differentiation 14
cf-mtDNA: Cell-free mitochondrial DNA
CK-MB: Creatine kinase-MB
cTnI: Cardiac troponin I
cTnT: Cardiac troponin T
DAMP: Damage-associated molecular pattern
ED: Emergency department
EV: Extracellular vesicle
ET-1: Endothelin-1
FN1: Fibronectin-1
H-FABP: Heart-type fatty acid-binding protein
ICU: Intensive care unit
IL-33: Nterleukin-33
LASSO: Least absolute shrinkage and selection operator
LDH: Lactate dehydrogenase
LGALS3: Galectin-3
ML: Machine learning
MR-proADM: Mid-regional pro-adrenomedullin
MYC: Myelocytomatosis oncogene
NOX: NADPH oxidase
NT-proBNP: N-terminal pro-B natriuretic peptide
PCT: Procalcitonin
PSP: Presepsin
RNA: Ribonucleic acid
SCM: Septic cardiomyopathy
SERPINE1: Plasminogen activator inhibitor-1
*sST2*: Soluble suppression of tumorigenicity-2
STAT3: Signal transducer and activator of transcription 3
TIMP1: Tissue inhibitor of metalloproteinases 1
TLR: Toll-like receptor
vWF: Von Willebrand factor
